# Learning Optimal Time-Frequency-Spatial Features by the CiSSA-CSP Method for Motor Imagery EEG Classification

**DOI:** 10.3390/s22218526

**Published:** 2022-11-05

**Authors:** Hai Hu, Zihang Pu, Haohan Li, Zhexian Liu, Peng Wang

**Affiliations:** Department of Precision Instrument, Tsinghua University, Beijing 100084, China

**Keywords:** motor imagery, circulant singular spectrum analysis (CiSSA), common spatial patterns (CSP), time-frequency-spatial features

## Abstract

The common spatial pattern (CSP) is a popular method in feature extraction for motor imagery (MI) electroencephalogram (EEG) classification in brain–computer interface (BCI) systems. However, combining temporal and spectral information in the CSP-based spatial features is still a challenging issue, which greatly affects the performance of MI-based BCI systems. Here, we propose a novel circulant singular spectrum analysis embedded CSP (CiSSA-CSP) method for learning the optimal time-frequency-spatial features to improve the MI classification accuracy. Specifically, raw EEG data are first segmented into multiple time segments and spectrum-specific sub-bands are further derived by CiSSA from each time segment in a set of non-overlapping filter bands. CSP features extracted from all time-frequency segments contain more sufficient time-frequency-spatial information. An experimental study was implemented on the publicly available EEG dataset (BCI Competition III dataset IVa) and a self-collected experimental EEG dataset to validate the effectiveness of the CiSSA-CSP method. Experimental results demonstrate that discriminative and robust features are extracted effectively. Compared with several state-of-the-art methods, the proposed method exhibited optimal accuracies of 96.6% and 95.2% on the public and experimental datasets, respectively, which confirms that it is a promising method for improving the performance of MI-based BCIs.

## 1. Introduction

Brain–computer interface (BCI) systems build a direct connection between the human brain and external devices, bypassing peripheral nerves and muscles [1]. BCIs not only help disabled patients effectively regain or recover motor function [2], but also have many promising applications for healthy users, such as gaming, car control [3], and fatigue detection [4]. Among BCI systems, motor imagery (MI)-based BCIs are more flexible than other types of BCIs because they can be driven by voluntary brain activities without external stimulation and can be more intuitive to control [5,6]. During MI, the sensorimotor rhythms are attenuated and then enhanced in a short time, which is known as event-related desynchronization/synchronization (ERD/ERS) [7]. Generally, the signal process of a MI EEG-based BCI system contains three stages: EEG signal recording, feature extraction, and classification. Among these, feature extraction is challenging due to non-stationarity and a low signal-to-noise ratio, which can affect the performance of MI-based BCIs [8].

To optimally extract EEG features that describe the ERD/ERS phenomenon, the common spatial pattern (CSP) algorithm, which seeks spatial filters to extract the class-discriminative spatial features [9], is frequently adopted due to its good performance. However, the performance of CSP is strongly affected by the frequency bands for sensorimotor rhythm extraction and the time period for analysis in the EEG signal [10]. Combining the spectral and temporal information in the CSP-based spatial feature is challenging. The effectiveness of CSP depends on identifying the optimal EEG frequency bands. Because the optimal frequency band is subject-specific, a fixed and broad frequency band (8–30 Hz), which is commonly used, is not suitable for all cases [11]. The classification accuracy may be decreased due to a poorly selected filter band that does not include sufficient spectral information [12]. To solve this issue, several extensions of CSP have been proposed to use narrowband information from different frequency bands, such as sub-band CSP (SBCSP) [13], filter bank CSP (FBCSP) [14], discriminative FBCSP (DFBCSP) [15], and sparse FBCSP (SFBCSP) [16]. These methods usually adopt a finite impulse response (FIR) filter or infinite impulse response (IIR) filter to obtain the sub-bands with different frequency bands, which cannot remove noise and artifacts overlapping in time–frequency space with the MI EEG signal [17]. These noise and artifacts decrease the classification accuracy of the CSP method.

In addition to frequency band optimization, another significant but often ignored issue in CSP is time window optimization for EEG segmentation that captures discriminative features [12]. An appropriate time window for EEG should be preselected to cover the significant ERD/ERS patterns when the imagery is activated and remove the unrelated time interval when the imagery is over. Many of the previous studies [11,16,18,19,20,21] have adopted a fixed and predefined time window (i.e., 0.5–2.5 s after the cue) for feature extraction of MI-related EEG. However, the optimal EEG time window varies over time and across subjects [22]. The use of a fixed time window can hardly capture discriminative temporal features for all subjects, and hence results in poor classification performance. In recent years, an increasing number of researchers have suggested that the optimization of the time window can significantly improve classification accuracy. Wang et al. introduced two Parzen window-based methods to select subject-specific time segments from 21 overlapping time window candidates [23]. Huang et al. [24], Miao et al. [6], and Kirar et al. [25] introduced methods that simultaneously optimize time windows and frequency sub-bands within the CSP to improve the performance of MI classification. Jin et al. designed a novel time filter that acted together with the spatial filter to introduce the temporal information in the spatial features, and discussed the effect of different lengths of time windows to obtain the optimal time segments [26]. Therefore, it is necessary to identify optimal and task-related frequency sub-bands and relevant time segments of EEG data to improve the performance of MI classification.

When frequency sub-bands and the time window are optimized, the time-frequency-spatial features from multichannel EEG recordings lead to a very high dimensional feature space. However, the high dimension feature space may contain irrelevant features [25] and have overfitting problems that inevitably diminish discriminative information [24]. These irrelevant and overfitting features decrease the performance of MI classification. In order to obtain a relevant subset of features and reduce the dimensionality of the feature space, the feature fusion method is used to obtain the optimal features. Feature fusion usually contains feature selection and dimensionality reduction. Feature selection is the process of finding the most effective features from the available feature set to improve algorithm performance. In the MI classification task, commonly used feature selection methods include L1-norm [18], Fisher score [27], mutual information [28], and neighborhood component analysis (NCA) [21]. Dimensionality reduction is a process of removing redundant variables, leaving the significant variables to improve the accuracy the classification tasks [29]. Principal component analysis (PCA) is a common and popular dimensionality reduction method.

In order to optimize the frequency band and time window for the combination of spectral and temporal information in the CSP-based spatial features, we propose a novel circulant singular spectrum analysis embedded CSP (CiSSA-CSP) method for learning the optimal time-frequency-spatial features to improve the classification accuracy of MI-related EEG. Prior to extracting features using CSP, the raw EEG data are segmented into multiple sub-time segments, from which spectrum-specific sub-bands are derived in a set of non-overlapping filter bands using CiSSA. The embedded CiSSA not only introduces additional spectral information in features, but suppresses noise and artifact overlapping in the frequency space with the EEG signal. Instead of adopting all the time-frequency-spatial features for classification, we devised a feature fusion based on mutual information or principal component analysis (PCA) to reduce redundant information and extract optimal CSP features. Thus, the MI classification accuracy is improved by the proposed method.

## 2. Methods

**Figure 1:**
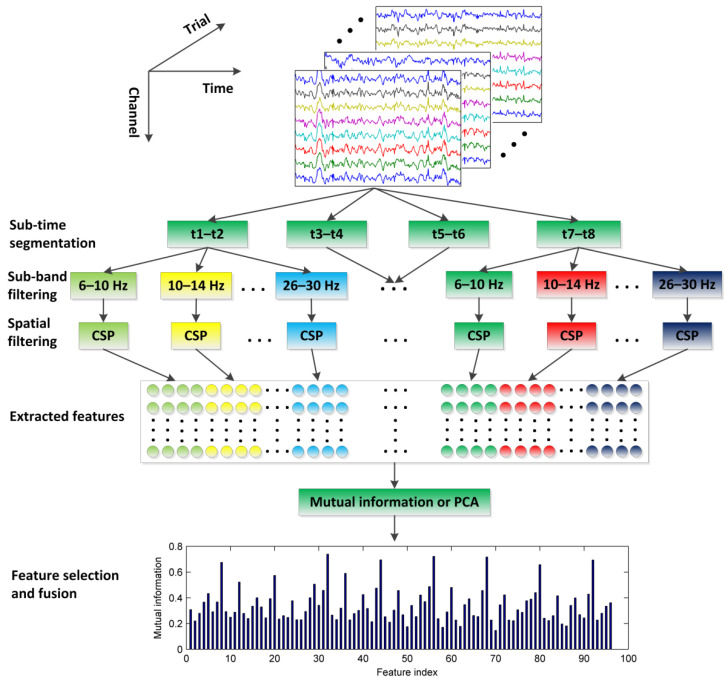
Illustration of the CiSSA-CSP method for motor-imagery classification.

The overall framework of the proposed CiSSA-CSP method for motor-imagery classification is illustrated in Figure 1, including time segmentation, sub-band filtering, CSP feature extraction, and feature fusion. Specifically, the multi-channel EEG signals are segmented into *T* = 4 segments with overlapping time using a sliding window. Every time segment is bandpass filtered into *B* = 6 sub-bands in a set of non-overlapping filter bands using the CiSSA method. Then, spatial features are extracted in every sub-band across all time segments by CSP, and the feature vector $F_{i}\in R^{2MBT}$ is obtained. Finally, the feature fusion method, including mutual information or PCA, is used to obtain the optimal features, which are then fed into the SVM for MI classification. A simple linear kernel and the constraint *C* = 1 is adopted for the SVM training.

### 2.1. Time Segmentation of EEG Signal

MI EEG signals have distinct temporal behavior and a transient nature [22]. CSP features extracted from the whole time period do not carry any temporal information as EEG signals are averaged over time to compute the covariance matrix. Therefore it is crucial to select the optimal time window and focus on the local properties of the EEG. In order to combine the temporal information in the CSP features, the raw EEG signals are segmented into *T* segments with overlapping time using a sliding window of length 2 s, which can discriminate different motor imagery stages.

### 2.2. Sub-Band Filtering Using CiSSA

In order to further combine the spectral information in the CSP features, the sub-bands are composed using the CiSSA method to perform bandpass filtering on all time segments. The CiSSA method is a nonparametric signal extraction method proposed by Juan Bógalo [30]. The CiSSA is derived from the singular spectrum analysis (SSA), which can suppress noise and artifacts with overlapping frequencies compared with the narrowband filter methods such as FIR and IIR [17]. It can decompose the signal into a set of reconstructed components (RCs) of known frequencies. CiSSA consists of four steps: embedding, decomposition, diagonal averaging, and grouping. In the time-delay embedding step, every single-channel EEG time series $s={\left(s_{1},s_{2},\ldots ,s_{N}\right)}^{T}$ (superscript *T* denotes the transpose of a vector) is mapped onto a multidimensional trajectory matrix **X** using a sliding window with the window length *L*. In the decomposition step, the trajectory matrix is decomposed into elementary matrices of rank 1 that are associated with different frequencies. To do so, a related circulant matrix $C_{L}$ is built based on the second order moments of the time series [30]:

(1) $$C_{L}(f)=\left(\begin{array}{ccccc} c_{0} & c_{1} & c_{2} & \cdots & c_{L-1}\\ c_{L-1} & c_{0} & c_{1} & \cdots & c_{L-2}\\ \vdots & \vdots & \vdots & \vdots & \vdots \\ c_{1} & c_{2} & c_{3} & \cdots & c_{0} \end{array}\right)$$

where:

(2) $$c_{m}=\frac{L-m}{L}\gamma_{m}+\frac{m}{L}\gamma_{L-m},\;\gamma_{m}=\frac{1}{N-m}\sum_{t=1}^{T-m}s_{t}s_{t+m},\;m=0,1,\ldots ,L-1$$

The eigenvalues and eigenvectors of $C_{L}$, respectively, are given by [31]:

(3) $$\begin{array}{l} \lambda_{k}=\sum_{m=0}^{L-1}c_{m}\exp(i2\pi m\frac{k-1}{L})=f(\frac{k-1}{L})\\ u_{k}=L^{-1/2}{\left(u_{k,1},u_{k,2},\ldots ,u_{k,L}\right)}^{H},\;k=1,2,\ldots ,L\\ u_{k,j}=\exp(-i2\pi (j-1)\frac{k-1}{L}),\;j=1,2,\ldots ,L \end{array}$$

where f(•) denotes the power spectral density of the signal. *H* indicates the conjugate transpose of a matrix. The *k*-th eigenvalue and corresponding eigenvector is associated with the specific frequencies given by:

(4) $$f_{k}=\frac{k-1}{L}f_{s}$$

where $f_{s}$ is the sampling rate of EEG signals.

Then, in the diagonal averaging step [32], several time series are reconstructed from the elementary matrices. The reconstructed time series are generally called RCs. Thus the raw EEG signal is decomposed into several RCs of known frequency given by Equation (4). The frequency bandwidth of each RC can be roughly expressed by [33]:

(5) $$f_{b}=f_{s}/L$$

As a consequence, the frequency bandwidth of each RC is limited to $f_{s}/L$. Considering the frequency of each RC given by Equation (4), there is no frequency mixing between different RCs.

We perform bandpass filtering on all time segments using the CiSSA method to obtain a set of non-overlapping sub-bands ($fb_{1},fb_{2},\ldots ,fb_{B}$). These sub-bands are chosen from the frequency range 6–30 Hz with bandwidth of $f_{b}=4\;Hz$, i.e., $fb_{1}=6–10\;Hz$, $fb_{2}=10–14\;Hz$, …, $fb_{B}=26–30\;Hz$ where *B* = 6. Then, feature extraction is performed on every sub-band using CSP.

### 2.3. Feature Extraction Using Common Spatial Patterns

Consider two classes of EEG signal $X_{i,1}$ and $X_{i,2}\epsilon R^{C\times P}$ recorded from the *i*-th trial, where *C* is the number of channels, and *P* denotes the number of sample points. The spatial covariance matrix **∑** of the class *l* (*l* = 1, 2) is given by:

(6) $$\Sigma_{l}=\frac{1}{N_{l}}\sum_{i=1}^{N_{l}}\frac{X_{i,l}X_{i,l}^{T}}{trace(X_{i,l}X_{i,l}^{T})}$$

where $N_{l}$ is the number of trials in class *l*. CSP aims at finding linear transforms (spatial filters) to maximize discrimination between two classes [16]. In order to achieve maximum separability between the variance of two classes, the Rayleigh quotient *J*(**w**) is introduced:

(7) $$\underset{w}{\max}J(w)=\frac{w^{T}\Sigma_{1}w}{w^{T}\Sigma_{2}w}\;s.t.\;||w|{|}_{2}=1$$

where $||●|{|}_{2}$ denotes the *l*_2_-norm and $w\in R^{C}$ is a spatial filter. The maximization of Rayleigh quotient J(w) can be achieved by solving the generalized eigenvalue problem: $\Sigma_{1}w=\lambda \Sigma_{2}w$. The learned linear transforms (spatial filters) matrix $W=[w_{1},w_{2},\cdots ,w_{2M}]$ can be obtained by collecting eigenvectors corresponding to the *M* largest and smallest generalized eigenvalues, which represent maximum discrimination between two classes. The spatial filtered EEG, which is the projection **Z** of EEG signal **X**, is then given by $Z=W^{T}X$.

The variance based CSP feature vector is then formed as $F=[F_{1},F_{2},\cdots ,F_{2M}]$, where *M* = 2, $F_{i}$ is given by [11]:

(8) $$F_{i}=\log(\mathrm{var}(Z_{i}))$$

where $\mathrm{var}(Z_{i})$ denotes the variance of *i*-th row of **Z**.

CSP is implemented on the segmented and filtered signals in each sub-band to calculate the corresponding features by Equation (8). As a result, 2*MBT* = 96 features are extracted from each EEG sample.

### 2.4. Feature Fusion

The method described above leads to a high-dimension feature set (dimension = 96) that is highly correlated. Obviously using all features for the final decision is not very efficient due to over-learning problems in high dimensions [13]. Therefore, dimension fusion steps are needed to reduce the feature dimensions and improve the performance of classification. We studied two common approaches to obtain a lower dimensionality subset and use them for final classification, namely, mutual information for feature selection and PCA for dimensionality reduction. We feed the reduced feature space to the support vector machine (SVM) and investigate the performance of EEG classification.

#### 2.4.1. Mutual Information

The mutual information-based individual feature (MIBIF) algorithm is a feature selection method that shows good performance in the CSP-based method [34]. For the feature vector set $F=\{F_{1},F_{2},\ldots ,F_{d}\},d=2MBT$, and the corresponding class label Ω={1,2}, the mutual information of each feature is calculated:

(9) $$I(F_{i};\Omega )=H(\Omega )-H(\Omega |F_{i}),i=1,2,\ldots ,d$$

where $H(\Omega )=-\sum_{\omega =1}^{2}p(\omega )\log_{2}p(\omega ),\omega \in \Omega$, the conditional entropy is:

(10) $$H(\Omega |F_{i})=-\sum_{\omega =1}^{2}p(\omega |F_{i})\log_{2}\;p(\omega |F_{i})$$

A higher magnitude of mutual information means more relevance between the feature and the class. Thus, the features are ranked in descending order according to mutual information and the top *k* significant features are selected.

#### 2.4.2. PCA

PCA is a useful approach to decorrelate the features and reduce the dimensionality of the feature space [35]. The purpose of PCA is to find the linear orthogonal transformation matrix that maximally maintains the feature variance [36]. The mean feature vector $m_{v}=\sum_{i=1}^{n}f_{i}/n$ is calculated from the feature vector set $F=[f_{1},f_{2},\cdots ,f_{d}]$, *d* denotes the number of features. Then, covariance matrix $C_{\mathrm{PCA}}$ for **F** is calculated as follows:

(11) $$C_{\mathrm{PCA}}=\frac{1}{n-1}\sum_{i=1}^{d}\left(f_{i}-m_{v}\right){\left(f_{i}-m_{v}\right)}^{T}$$

The PCA projection matrix $W_{\mathrm{PCA}}$ can be obtained by calculating the eigenvectors and eigenvalues for the covariance matrix and selecting the top *k* columns of eigenvectors in descending order of eigenvalue sizes.

## 3. Data and Experiment

In order to better verify the validity of the proposed CiSSA-CSP method, we used two different MI EEG datasets for analysis. The first dataset was the BCI Competition III dataset IVa, which is publicly available and has been used in many studies [34,37]. Therefore, using this dataset, we can effectively compare our method with competing methods. In addition, in order to verify the universal applicability of the method, the second dataset, which was collected by ourselves, was used for analysis and validation.

### 3.1. Public EEG Dataset

**Figure 2:**
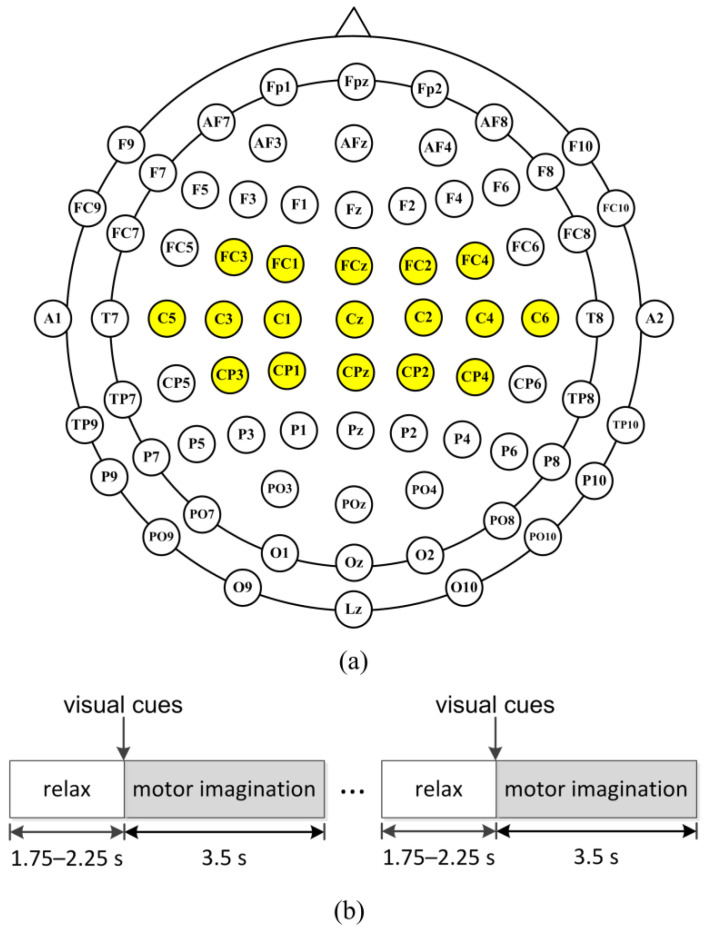
(**a**) Electrodes used in our study (yellow circles) according to the extended international 10–20 system. (**b**) The scheme of the experiment. A single trial of the experiment was divided into two periods. In the first period, the subject relaxed for 1.75–2.25 s; and then the visual cues were indicated for 3.5 s when the subject performed the motor imageries.

BCI Competition III dataset IVa was recorded from five healthy subjects (subject aa, al, av, aw, and ay). The subjects sat in a comfortable chair and performed motor imagery (right hand and right foot) experiments. The EEG signal was recorded using 118 channels according to the extended international 10–20 system and 140 trials for each class. Thus, a total of 280 trials were provided for each subject. The sampling rate of the EEG data was 100 Hz. Each trial lasted 3.5 s of motor imagery and was interrupted by a time period of 1.75 to 2.25 s, in which the subject could relax, shown in Figure 2b. Seventeen EEG channels were selected in our study, as shown in Figure 2a (FC3, FC1, FCz, FC2, FC4, C5, C3, C1, Cz, C2, C4, C6, CP3, CP1, CPz, CP2, CP4), which contain the sensorimotor area needed to recognize the cue in the experiment [34].

### 3.2. Experimental EEG Dataset

**Figure 3:**
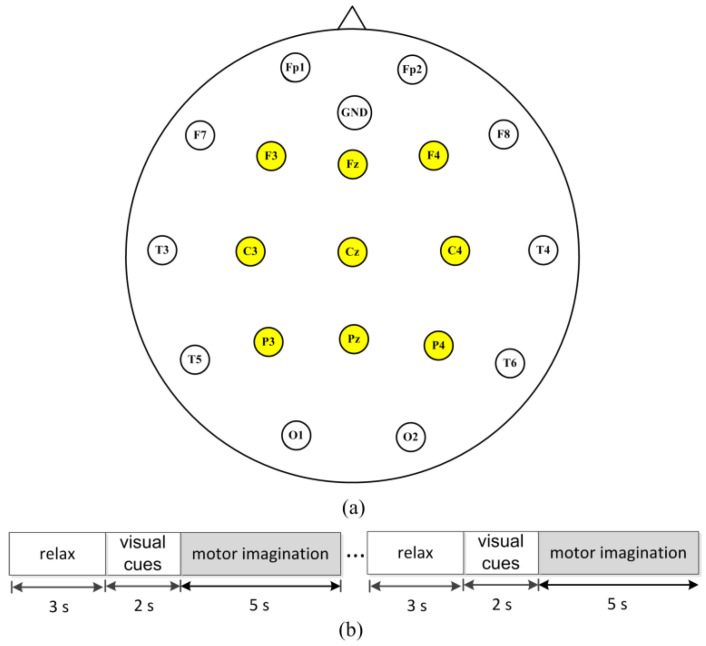
Experiment setup. (**a**) Electrodes used in the experiment (yellow circles) according to the international 10−20 system. (**b**) The scheme of the experiment. A single trial of the experiment was divided into three periods. In the first period, the subject relaxed for 3 s; and then the visual cues were indicated for 2 s for preparation. Finally, subjects performed the motor-imagery tasks (right hand or foot) for 5 s.

The experiments were approved with a protocol (NO. 20170010) by the Institutional Review Board of Tsinghua University and written informed consent was obtained from the subjects. Twenty healthy subjects (subject S1, S2, …, S20) aged 20–29 participated in the experiments and abstained from psychoactive substances for at least 4 h prior to the experiments. The experiments were carried out with the subjects sitting on a comfortable chair in a room with normal lightness. The experimental EEG signals were recorded with nine electrodes (F3, Fz, F4, C3, Cz, C4, P3, Pz, P4) from the international 10–20 system, shown in Figure 3a, using the MP160 data acquisition and analysis system (BIOPAC Systems, Inc., Goleta, CA, USA). During each trial, as shown in Figure 3b, the subject relaxed for 3 s, and then a visual cue was presented. Two seconds later, the subject performed the right-hand or right-foot motor imaginary tasks for 5 s. There were 140 trials for each class per subject, i.e., a total of 280 trials for each subject. All EEG signals were recorded at a sampling rate of 250 Hz.

## 4. Results and Discussion

### 4.1. Results and Discussion of Public EEG Dataset

The 17-channel EEG signals of all trials were segmented into *T* = 4 segments with overlapping time of 1.5 s (0–2 s, 0.5–2.5 s, 1–3 s, 1.5–3.5 s). Then, every time segment was bandpass filtered into *B* = 6 sub-bands without overlapping frequency using the CiSSA method (6–10 Hz, 10–14 Hz, 14–18 Hz, 18–22 Hz, 22–26 Hz, 26–30 Hz). A total of 2*M* = 4 features were extracted from every sub-band by CSP and, thus, 2*MBT* = 96 features were obtained. Finally, dimension reduction was conducted by mutual information or PCA and optimal features are selected for MI classification. A 10-fold cross-validation was implemented to evaluate the classification performance.

Table 1 shows the classification accuracy of different algorithms for five subjects using 10-fold cross-validation. The classification performance of standard CSP is not very good, especially for subject aa, av, and aw. The average classification accuracy of CSP is 81.6%. We refer to classification results obtained with CiSSA filtered sub-bands before CSP as CiSSA + CSP. Classification results indicated that CiSSA + CSP provides improvements compared to CSP for all subjects, especially for aa, av, and aw. The average classification accuracy of CiSSA + CSP is 92.3%, which is much higher than the accuracy of CSP. This proves that combining spectral information in the CSP features can greatly improve the performance of MI classification. The results corresponding to the Subtime + CiSSA + CSP are obtained with time segmentation processing before CiSSA + CSP. With the exception of subject aw, the classification accuracy increases with all subjects when time segmentation is implemented. The average accuracy of Subtime + CiSSA + CSP increases to 94.5%, proving that combining temporal information in the CSP features can further improve the classification accuracy of MI EEG. The results obtained with MIBIF and PCA processing as dimensionality reduction are referred as Subtime + CiSSA + CSP + MIBIF and Subtime + CiSSA + CSP + PCA, respectively. Note that *k* = 9 optimal features are selected for all subjects to preliminary study the effects of MIBIF and PCA. When MIBIF is used as the feature selection method, the classification accuracy decreases for subjects aa, al, and av, and the average classification accuracy decreases to 93.6%. This indicates that nine optimal features are not enough to carry sufficient discriminative information. Further studies should be conducted to select suitable and optimal features. When PCA is used for dimensionality reduction, the classification accuracy increases slightly with all subjects except for subject aa. Subtime + CiSSA + CSP + PCA provides the best results with an average classification accuracy of 96.4%.

#### 4.1.1. Discriminative Frequency Sub-Band Features

**Figure 4:**
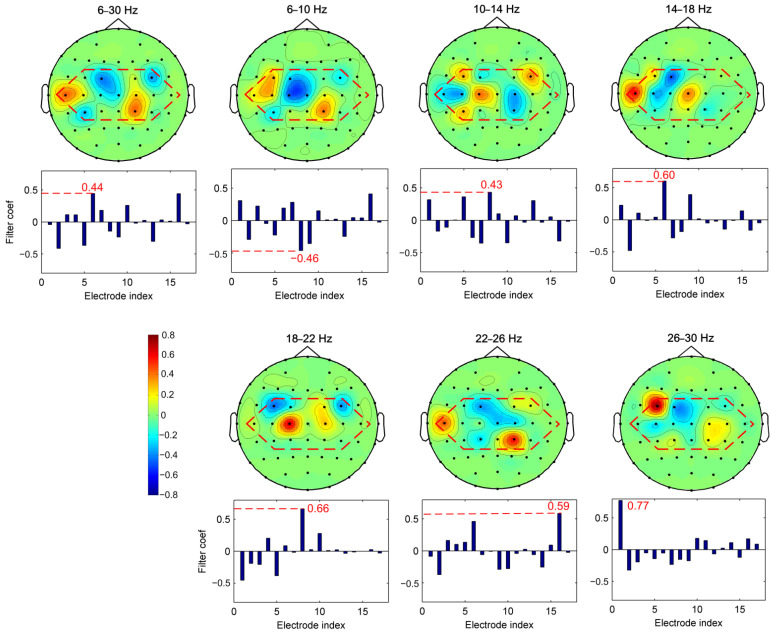
The topographical map and the filter coefficient of the most significant spatial filter learned by the CSP method of each sub-band for subject av. The electrode indexes 1, 2, …, 17 correspond to the electrode FC3, FC1, FCz, FC2, FC4, C5, C3, C1, Cz, C2, C4, C6, CP3, CP1, CPz, CP2, CP4, respectively. Electrodes inside the red outline represent the electrode indexes 1, 2, …, 17.

In order to understand the effect of the combination of spectral information with CSP features, we visualized the topographical distribution of the broad band and the sub-band EEGs. Figure 4 presents the topographical map and the filter coefficient of the most significant spatial filter learned by the CSP method from the broad band and all sub-bands for subject av. Only the electrodes of the sensorimotor area (inside the red dotted frame) are presented in the topographical map. A larger absolute value of the filter coefficient means more discriminative information [16]. It can be seen that the largest filter coefficient of the most significant spatial filter in the broad frequency band (6–30 Hz) is only 0.44, which leads to poor separability. The largest filter coefficients of the most significant spatial filter in sub-bands 6–10 Hz, 10–14 Hz, 14–18 Hz, 18–22 Hz, 22–26 Hz, and 26–30 Hz are −0.46, 0.43, 0.6, 0.66, 0.59 and 0.77, respectively. This indicates better separability in Beta rhythm sub-bands (14–18 Hz, 18–22 Hz, 22–26 Hz, and 26–30 Hz) than in Mu rhythm sub-bands (6–10 Hz and 10–14 Hz) and broad band (6–30 Hz) for subject av. More discriminative spectral information is combined in the Beta rhythm sub-bands features. Therefore, we need to find more precise frequency sub-bands for MI CSP feature extraction, since these sub-bands carry the most discriminative information and the remaining sub-bands are irrelevant and redundant to the MI tasks. It is concluded that the combination of spectral information in the CSP features by an effective optimization of filter band is necessary to improve the MI classification performance.

**Figure 5:**
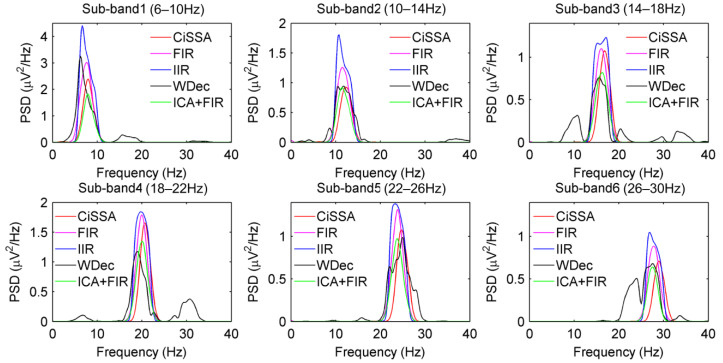
The power spectrum density (PSD) of the sub-bands extracted by CiSSA, FIR, IIR, WDec, and ICA + FIR for subject av at electrode C3. The PSDs of sun-bands extracted by FIR and IIR are higher than those by CiSSA and ICA + FIR. The PSDs of sun-bands extracted by WDec contain components falling outside the frequency width (e.g., 6–10 Hz for sub-band1).

In the study, the sub-bands of the EEG were extracted by the CiSSA. In order to compare the performance of sub-band extraction and classification with other common filtering methods, the sub-bands were extracted by FIR filtering with order 60, Butterworth IIR filtering with order 7, and the wavelet decomposition (WDec) methods, and the classification accuracies were calculated on CSP features extracted from these sub-bands. Furthermore, the independent component analysis (ICA) method is commonly used in signal decomposition and artifact removal of EEG [38]. The noise and artifacts are removed by the FastICA method based on Negentropy [38] and then the sub-bands are extracted by FIR filtering. Table 2 shows the classification accuracies of CSP, FIR + CSP, IIR + CSP, WDec + CSP, ICA + CSP, ICA + FIR + CSP, and CiSSA + CSP methods on BCI Competition III dataset IVa. It can be seen that, compared with the standard CSP on the broad EEG band, the classification accuracies are highly improved for sub-bands by FIR, IIR, WDec and CiSSA. This proves that combining spectral information can greatly improve the discrimination of CSP features. Expert for subject ay, the classification accuracies obtained by CiSSA are higher than those obtained by FIR, IIR, and WDec for all subjects. The average classification accuracy obtained by CiSSA is improved by 2.3%, 2.9%, and 1.9% over the average classification accuracies obtained by FIR, IIR, and WDec, respectively. This is because the CiSSA can suppress noise and artifacts with overlapping frequencies of sub-bands, while the FIR, IIR, and the WDec methods are not able to separate the noise overlapping in the frequency space, which decreases the classification performance of CSP. Figure 5 shows the power spectrum density (PSD) of the sub-bands extracted by CiSSA, FIR, IIR, WDec, and ICA + FIR for subject av at electrode C3. It can be seen that the PSDs of sub-bands extracted by FIR and IIR are higher than those by CiSSA and ICA + FIR, which can suppress noise and artifacts with overlapping frequencies. The PSDs of sub-bands extracted by WDec contain components falling outside the frequency width of sub-bands. Although ICA can also remove noise and artifacts with overlapping frequencies, the average classification accuracy obtained by ICA + FIR is lower than that obtained by CiSSA. It is concluded that the CiSSA extracts more precise frequency sub-bands for MI CSP feature extraction.

The bandwidth of sub-bands is 4 Hz, which is used in most of the previous studies [8,10,12,24,39]. Table 3 shows the classification accuracies of CiSSA + CSP method on different bandwidths on BCI Competition III dataset IVa using 10-fold cross-validation. It can be seen that the classification accuracy attains a high value when the bandwidth is set to be 1 or 4 Hz. However, more computing resources and time are needed for a bandwidth of 1 Hz than for a bandwidth of 4 Hz. Therefore, the bandwidth of 4 Hz is the best choice for sub-band extraction.

#### 4.1.2. The Performance of Time Segmentation

**Figure 6:**
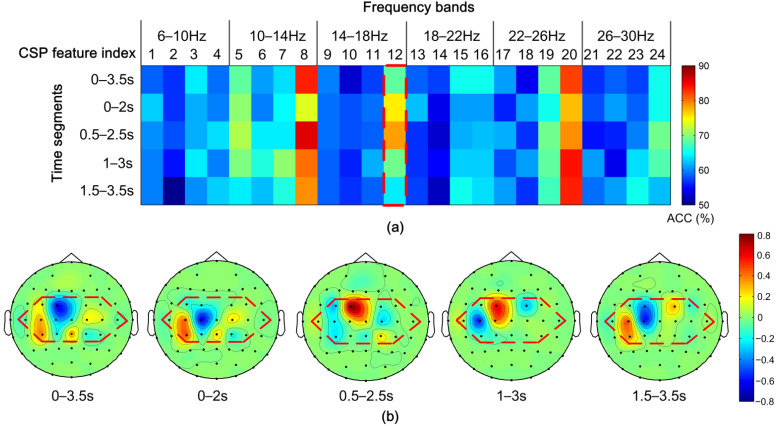
Performance of time segmentation for subject aa. (**a**) Pictorial representation of the classification accuracy (ACC) on the feature space learned by the proposed method for subject aa. Each time-frequency segment contains 4 CSP features. (**b**) The topographical maps of the most significant spatial filter learned by the CSP from all time windows in sub-band 14–18 Hz (marked by red outline in Figure 6a). Electrodes inside red outline in Figure 6b represent the electrodes of the sensorimotor area.

To present time window segmentation performance, we made topoplots of spatial filters for subject aa as an example, shown in Figure 6. Figure 6a shows the classification accuracy of the feature space learned by the proposed CiSSA-CSP method using a pictorial representation. Overall, the feature space has five time windows (the whole time window and four sub-time windows) and six frequency sub-bands for each time window. Each time-frequency segment contains four CSP features. It can be observed that CSP feature index 8 (sub-band 10–14 Hz), index 12 (sub-band 14–18 Hz), and index 20 (sub-band 22–26 Hz), which represent the most significant spatial filters learned by the CSP from the sub-bands, attain the best classification accuracy. Features from CSP feature index 12 in all time windows are marked by a red outline. The classification accuracy of sub-time window 0.5–2.5 s is higher than the accuracies of the whole time window of 0–3.5 s and other sub-time windows of 0–2 s, 1–3 s, and 1.5–3.5 s. To further analyze the effect of the proposed method in different time windows, the topographical maps of the most significant spatial filter learned by the CSP from all time windows in sub-band 14–18 Hz (marked by red outline in Figure 6a) are shown in Figure 6b. An evident change in ERD/ERS patterns in the sensorimotor area is observed as the time window changes, which shows that the neural response during motor imagery tasks changes with time. In sub-time windows 0.5–2.5 s, spatial features are more discriminative and significant than in other sub-time windows and the whole time window. Therefore, combining temporal information into CSP features by time segmentation leads to more discriminatory features for MI task classification.

The performance of classification is affected by time-window length. The classification accuracies at different time-window lengths with a window step of 0.5 s were calculated using 10-fold cross-validation and the results are shown in Table 4. It can be seen that the classification accuracies vary within a small range of 1.2% and the accuracy attains a maximum value at time-window length of 2 s.

#### 4.1.3. The Effect of Feature Selection by MIBIF

**Figure 7:**
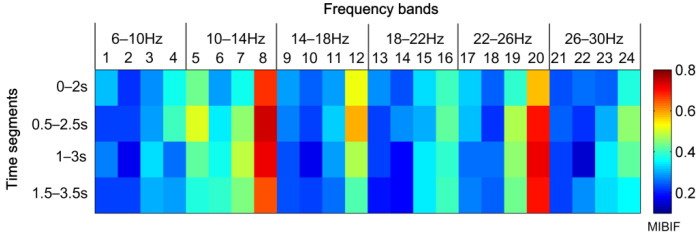
Distribution of MIBIF values in all time-frequency segments for subjects aa. Index 1, 2, …, 24 in the frequency bands represent the CSP feature index.

**Figure 8:**
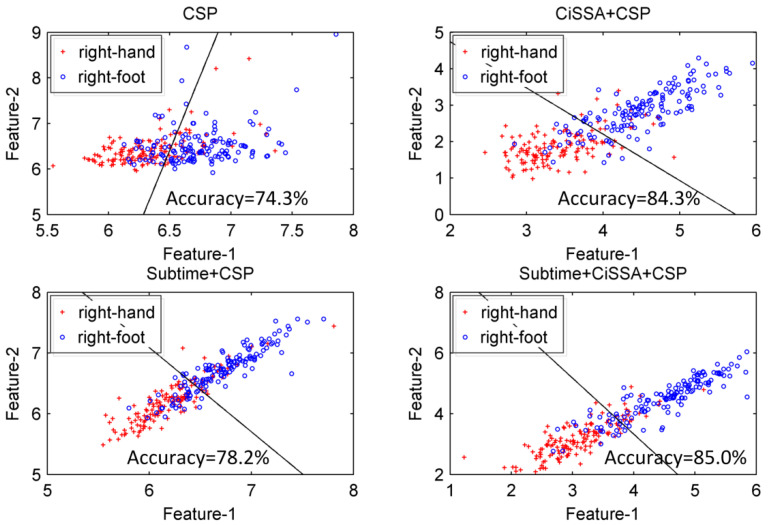
Distributions of the most two significant features obtained by CSP, CiSSA + CSP, Subtime + CSP and Subtime + CiSSA + CSP, for subjects aa.

To have an intuitive understanding of the distribution of significant time-frequency segments, the values of MIBIF belonging to each time-frequency segment were calculated, as shown in Figure 7 for subject aa. It can be seen that the highest values are located in feature indexes 8, 12, and 20, which is consistent with Figure 6. It is concluded that the features of higher MIBIF values contain more discriminatory information for accuracy improvement of MI EEG. In addition, the significance (MIBIF value) changes along the time axis and frequency bands due to the non-stationarity of EEG. The most significant features are located in some local time-frequency segments. Therefore, it is believed that decomposing a multi-channel EEG into time-frequency segments for more precise analysis helps to improve the classification accuracy. Figure 8 depicts distributions of the most significant two features derived by CSP, CiSSA + CSP, Subtime + CSP, and Subtime + CiSSA + CSP, for subject aa. It is indicated that when the spectral (CiSSA + CSP) or temporal (Subtime + CSP) information is combined into the CSP features, more separable feature distributions are provided in comparison with the standard CSP. The highest discriminability of features was achieved by Subtime + CiSSA + CSP, which combines both the spectral and temporal information. The classification accuracy of the two most significant features derived by Subtime + CiSSA + CSP is 85.0%, 10.7% higher than the classification accuracy obtained by standard CSP.

**Figure 9:**
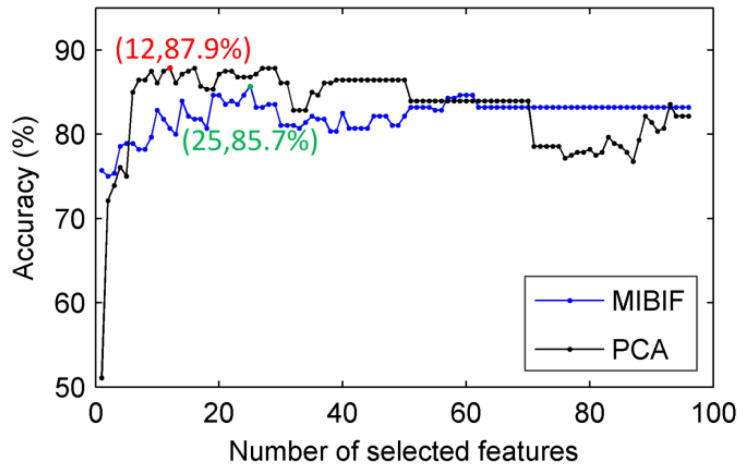
Classification accuracy over the number of selected features by MIBIF and PCA for subjects av.

The classification accuracy of the proposed mothed varies with the number of the features selected by the MIBIF. To select the most suitable features, the classification accuracies for the number of selected features by MIBIF were calculated, as shown in Figure 9 for subject av. Figure 9 indicates that the highest classification accuracy (85.7%) is attained when the most significant 25 features are selected for subject av. The highest classification accuracies and the number of selected features by MIBIF for all subjects are shown in Table 5. The average highest classification accuracy with feature selection by MIBIF for all subjects is 96.3%, which is a 1.4% improvement compared to the average classification accuracy without feature selection.

#### 4.1.4. The Effect of Dimensionality Reduction by PCA

**Figure 10:**
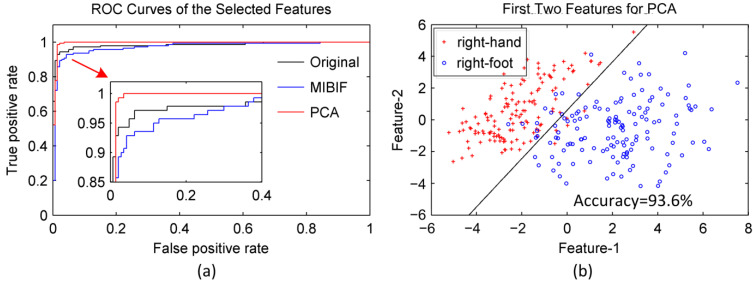
(**a**) The ROC curve of the 57 features selected by MIBIF and 5 features selected by PCA for subjects aa. (**b**) The distribution of the first two features obtained by PCA for subject aa. Note that the right-hand (blue, circle) and right-foot (red, cross) imagery classes are nearly linearly separable with only 2 features.

We note from Table 1 that the classification accuracies are increased when features are dimensionally reduced by PCA for certain subjects (av, aw, and ay). The receiver operating characteristic (ROC) curves related to the 57 features selected by MIBIF and five features selected by PCA for subject aa are given in Figure 10a. It is indicated that the area under the PCA curve is greater than the areas under curves of selected MIBIF features and all the original features, which means more discrimination in the selected features by PCA than by MIBIF. Figure 10b shows the distribution of the first two features obtained by PCA for subject aa. Note that the right-hand and -foot imagery classes are nearly linearly separable with the top two features with PCA. The classification accuracy of the first two features derived by PCA is 93.6%, higher than the classification accuracy derived by MIBIF (shown in Figure 8).

**Figure 11:**
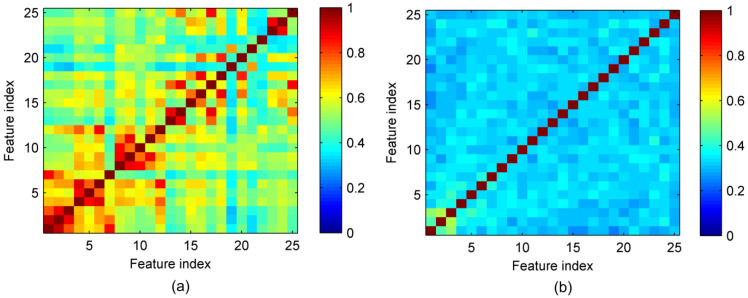
The distribution of mutual information between the top 25 features selected by (**a**) MIBIF and (**b**) PCA for subject av.

Similar to MIBIF, the classification accuracy also varies with the feature dimension selected by the PCA. It is indicated by Figure 9 that the highest classification accuracy (87.9%) is attained when the most significant 12 features derived by PCA are selected for subject av. The classification accuracy of PCA is higher than that of MIBIF when the same number of top significant features is selected. This is because PCA can decorrelate the features and reduce the redundant information between features, while MIBIF extracts features most relevant to the class. Features extracted by MIBIF may be highly correlated and contain redundant information. Figure 11 shows the distribution of mutual information between top 25 features selected by MIBIF and PCA for subject av. A higher value of mutual information means more relevance between two features. It can be seen that the top features extracted by MIBIF are highly correlated, while the features extracted by PCA are not correlated. The highest classification accuracies and the selected feature dimension by PCA for all subjects are shown in Table 5. The average highest classification accuracy with dimensionality reduction by PCA for all subjects is 96.6%, 0.3% higher than the accuracy derived by MIBIF.

#### 4.1.5. Comparison with Other Competing Techniques

Since accuracy is the key criterion for evaluating the performance of methods in a BCI system, we compared the classification accuracy of the proposed CiSSA-CSP method with other competing methods. Table 6 provides a comparative study of the classification performance between the proposed method and ten recently reported methods for Competition III dataset IVa, namely, FBCSP [14], CTFSP [6], Fusion [18], TWFBCSP-MVO [24], SFBCSP [16], STFSCSP [39], DFBCSP [40], CC-LR [37], ISSPL [41], and Class Separability (CS) [35] methods. The highest classification accuracies among these methods are highlighted in bold font for each subject and their averages. The highest classification accuracy of our proposed method is 100% for subject aw. Furthermore, the classification accuracies of our proposed method for subjects aa, al, and ay are very close to the highest classification accuracy of other competing methods. The average classification accuracies of our proposed method are 96.3% and 96.6% for MIBIF and PCA feature selection, respectively, which are higher than the average classification accuracies of other methods. It can be concluded that the proposed method outperforms the recently reported competing methods for MI EEG classification.

#### 4.1.6. Computational Complexity

**Figure 12:**
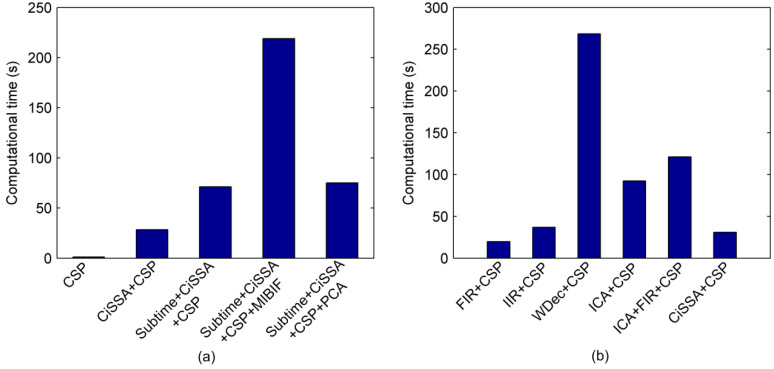
Computational time taken by different methods on Competition III dataset IVa with 10-fold cross-validation. (**a**) Computational time taken by CSP, CiSSA + CSP, Subtime + CiSSA + CSP, Subtime + CiSSA + CSP + MIBIF and Subtime + CiSSA + CSP + PCA. (**b**) Computational time taken by FIR + CSP, IIR + CSP, WDec + CSP, ICA + CSP, ICA + FIR + CSP and CiSSA + CSP.

In order to investigate the computational complexity of the proposed method, we calculated the time consumption of training and testing phase on Competition III dataset IVa. The experiment was implemented using MATLAB R2014a on a PC with Intel(R) Core(TM) 2.40 GHz CPU and 8.0 GB RAM. Figure 12 shows the computational time of the training phase taken by different methods with 10-fold cross-validation. From Figure 12a, it can be seen that combining spectral and temporal information in the CSP features by sub-band filtering (CiSSA + CSP) and time segmentation (Subtime + CiSSA + CSP) takes much more time than the CSP method. In addition, the time required by the MIBIF feature selection method is much longer than PCA. Furthermore, we compared the time consumption of CiSSA and other common filtering methods, as shown in Figure 12b. The results indicate that CiSSA and FIR consume the least time, while CiSSA achieves the highest classification accuracy (shown in Table 2).

After training, the optimal CSP filter for each time-frequency segment, the indexes of selected features, and the SVM model can be directly used for testing. Hence the computational time is significantly reduced during the testing phase. Table 7 lists the average testing time of one trial using our method and other recently reported methods for subject aa. The results indicate that, for one test trial, the average execution time of our method is 156.4 ms (MIBIF) or 156.7 ms (PCA). Although our method takes a longer time to compute one trial than other competing methods, it can meet the requirement of real-time processing since the computational time is much less than the length of one trial (3.5 s). Therefore, the proposed method improves the motor imagery classification performance without degrading the computation efficiency for BCI applications.

### 4.2. Results and Discussion of Experimental EEG Dataset

In the study of the experimental EEG dataset, the 9-channel EEG signals of all trials were segmented into *T* = 4 epochs with overlapping time of 1s (0–2 s, 1–3 s, 2–4 s, 3–5 s). Table 8 shows the classification accuracies of different algorithms for twenty subjects using 10-fold cross-validation. The classification performance of CSP is poor for most subjects and the average accuracy of CSP is 74.7%. When spectral information is combined with the CSP features by decomposing the EEG into sub-bands using CiSSA, the classification performance improves compared to CSP for all subjects. The average classification accuracy of CiSSA + CSP is 90.4%. The average accuracy of Subtime + CiSSA + CSP further increases to 92.3%. Similar to the public available dataset, *k* = 9 optimal features were selected for all subjects to preliminarily study the effects of MIBIF and PCA. When MIBIF is used as the feature selection method after Subtime + CiSSA + CSP, the classification accuracy decreases for most subjects and the average classification accuracy decreases to 89.8%, indicating that nine optimal features are not enough to carry sufficient discriminative information. When PCA is used for dimensionality reduction after Subtime + CiSSA + CSP, the classification accuracy increases slightly with all subjects, except for subjects S1, S12, and S14. Subtime + CiSSA + CSP + PCA provides the best results with an average classification accuracy of 93.9%. The results of the experimental EEG dataset are consistent with the results of Competition III dataset IVa. It is concluded that the proposed CiSSA-CSP method can be used in different MI datasets, which verifies the universal applicability of the method. To verify the reliability of the experimental results, a paired *t*-test [27] is used between two adjacent methods in Table 1 and Table 8 to show the statistical difference in the classification accuracies of different methods. The paired *t*-test’s results on all subjects of public and experimental datasets are shown in Table 9. It can be seen that all the *p*-values are less than 0.05 (*p* < 0.05), which means all improvements are statistically significant.

The classification accuracy of the proposed mothed on the experimental EEG dataset varies with the number of features selected by MIBIF or PCA. To select the most suitable features, the classification accuracies over the number of selected features by MIBIF or PCA were calculated, and we chose the number of features having the highest accuracy. Table 10 shows the highest classification accuracies and the selected feature dimension by MIBIF and PCA. It can be seen that, for all subjects except for subject S11, the number of features selected by PCA is smaller than that by MIBIF, while the classification accuracy derived by PCA is higher than that of MIBIF. The average highest classification accuracy with dimensionality reduction by PCA for all subjects is 95.2%, 1.5% higher than the accuracy derived by MIBIF.

## 5. Conclusions

We propose a novel algorithm, CiSSA-CSP, for learning the optimal time-frequency-spatial patterns to improve classification accuracy of MI EEG. Specifically, raw EEG data are first segmented into multiple time segments using a sliding window. Spectrum-specific sub-bands are further derived for each time segment in a set of non-overlapping filter bands using CiSSA. Therefore, features extracted in all time-frequency segments using CSP combine more sufficient and discriminative time-frequency-spatial information. We then devised a feature fusion based on mutual information or PCA to extract robust and optimal CSP features. A linear SVM classifier was trained on the optimized EEG features to accurately identify the MI tasks. The experimental study implemented on the public and experimental EEG datasets validated the effectiveness of the CiSSA-CSP method. Compared with several other competing methods, the proposed CiSSA-CSP method leads to a superior classification accuracy (averaged classification accuracies were 96.6% and 95.2% for the public and experimental datasets, respectively), which confirms that it is a promising method for improving the performance of MI-based BCIs.

## Figures and Tables

**Table 1:** The classification accuracies of the proposed CiSSA-CSP method on BCI Competition III dataset IVa (subject aa, al, av, aw, and ay).

Method	Classification Accuracy (%)					
	aa	al	av	aw	ay	Average
CSP	78.6 ± 11.4	96.4 ± 3.8	69.6 ± 10.7	75.0 ± 6.3	88.6 ± 5.0	81.6 ± 7.4
CiSSA + CSP	94.3 ± 5.9	98.2 ± 3.5	78.6 ± 6.5	98.2 ± 2.5	92.4 ± 4.1	92.3 ± 4.5
Subtime + CiSSA + CSP	98.6 ± 1.8	99.3 ± 1.5	83.2 ± 6.1	97.9 ± 3.0	95.7 ± 2.8	94.9 ± 3.0
Subtime + CiSSA + CSP + MIBIF	94.3 ± 6.6	98.2 ± 1.9	79.6 ± 7.4	98.2 ± 2.5	97.9 ± 3.8	93.6 ± 4.4
Subtime + CiSSA + CSP + PCA	98.2 ± 3.0	99.3 ± 1.5	87.5 ± 7.6	100 ± 0	97.1 ± 2.8	96.4 ± 3.0

**Table 2:** The classification accuracies of FIR + CSP, IIR + CSP, WDec + CSP, ICA + CSP, ICA + FIR + CSP, and CiSSA + CSP methods on BCI Competition III dataset IVa (subject aa, al, av, aw, and ay).

Method	Classification Accuracy (%)					
	aa	al	av	aw	ay	Average
FIR + CSP	85.7 ± 8.8	95.4 ± 3.8	78.6 ± 8.8	97.1 ± 2.3	93.2 ± 4.6	90.0 ± 5.7
IIR + CSP	87.1 ± 9.9	93.9 ± 4.1	76.8 ± 12.3	97.9 ± 3.0	91.4 ± 4.5	89.4 ± 6.8
WDec + CSP	93.9 ± 8.4	96.8 ± 3.6	72.6 ± 10.4	97.9 ± 3.8	90.7 ± 4.2	90.4 ± 6.1
ICA + CSP	81.1 ± 6.5	95.0 ± 5.1	71.1 ± 10.0	77.5 ± 6.1	94.3 ± 3.5	83.6 ± 6.2
ICA + FIR + CSP	90.4 ± 8.1	93.6 ± 2.8	81.1 ± 7.7	94.3 ± 3.8	95.7 ± 2.3	91.0 ± 4.9
CiSSA + CSP	94.3 ± 5.9	98.2 ± 3.5	78.6 ± 6.5	98.2 ± 2.5	92.4 ± 4.1	92.3 ± 4.5

**Table 3:** The classification accuracies of CiSSA + CSP methods on different bandwidths on BCI Competition III dataset IVa (subject aa, al, av, aw, and ay).

Bandwidth (Hz)	*L*	Classification Accuracy (%)					
		aa	al	av	aw	ay	Average
1	100	93.5 ± 4.1	98.2 ± 2.5	84.3 ± 7.3	91.0 ± 4.8	94.1 ± 4.7	92.2 ± 4.7
2	50	88.3 ± 6.6	97.4 ± 2.3	79.6 ± 6.7	96.4 ± 2.8	92.3 ± 6.3	90.8 ± 4.9
4	25	94.3 ± 5.9	98.2 ± 3.5	78.6 ± 6.5	98.2 ± 2.5	92.4 ± 4.1	92.3 ± 4.5
6	16	90.7 ± 6.1	97.5 ± 2.9	78.9 ± 11.0	97.1 ± 2.8	94.3 ± 4.8	91.7 ± 5.5
8	12	88.6 ± 9.3	98.6 ± 1.8	73.6 ± 9.7	92.9 ± 4.8	92.5 ± 3.9	89.2 ± 5.9

**Table 4:** The classification accuracies at different time-window lengths on BCI Competition III dataset IVa (subject aa, al, av, aw, and ay).

Time-Window Length (s)	Classification Accuracy (%)					
	aa	al	av	aw	ay	Average
1	98.3 ± 2.4	100	79.9 ± 3.8	96.1 ± 1.7	94.3 ± 2.4	93.7 ± 2.1
1.5	96.5 ± 2.5	99.6 ± 1.1	85.3 ± 5.4	97.6 ± 1.1	94.3 ± 1.5	94.7 ± 2.3
2	98.6 ± 1.8	99.3 ± 1.5	83.2 ± 6.1	97.9 ± 3.0	95.7 ± 2.8	94.9 ± 3.0
2.5	96.8 ± 2.6	99.0 ± 1.1	82.5 ± 8.0	97.9 ± 3.0	91.1 ± 5.1	93.5 ± 4.0
3	97.1 ± 2.8	99.0 ± 1.5	81.1 ± 6.1	97.5 ± 3.4	92.9 ± 6.1	93.5 ± 4.0

**Table 5:** The highest classification accuracies and the selected feature dimension (*k*) by MIBIF and PCA for all subjects on Competition III dataset IVa (subject aa, al, av, aw, and ay).

Subject	MIBIF		PCA	
	Accuracy (%)	Dimension (*k*)	Accuracy (%)	Dimension (*k*)
aa	98.6 ± 1.8	57	98.2 ± 2.5	5
al	99.6 ± 1.1	28	99.6 ± 1.1	11
av	85.7 ± 7.9	25	87.9 ± 6.8	12
aw	99.6 ± 1.1	10	100	9
ay	97.9 ± 3.8	8	97.5 ± 4.7	16
Average	96.3 ± 3.1		96.6 ± 3.0	

**Table 6:** Comparison of the classification performance between the proposed method and eight recently reported methods for Competition III dataset IVa (subject aa, al, av, aw, and ay).

Method	Classification Accuracy (%)					
	aa	al	av	aw	ay	Average
FBCSP [14]	83.6	94.6	51.4	93.9	88.2	82.4
CTFSP [6]	86.1	98.6	52.1	96.1	92.1	85.0
Fusion [18]	80.0	96.8	70.0	92.5	91.1	86.1
TWFBCSP-MVO [24]	89.6	99.3	69.3	96.1	92.1	89.3
SFBCSP [16]	91.5	98.6	77.4	98.0	94.7	92.0
STFSCSP [39]	92.5	98.6	79.4	97.8	95.0	92.7
DFBCSP [40]	92.3	99.3	78.1	99.3	95.1	92.8
CC-LR [37]	**100**	94.2	**100**	**100**	75.3	93.9
ISSPL [41]	93.6	**100**	79.3	99.6	**98.6**	94.2
Class Separability [35]	95.6	99.7	90.5	98.4	95.7	96.0
Our method (MIBIF)	98.6	99.6	85.7	99.6	97.9	96.3
Our method (PCA)	98.2	99.6	87.9	**100**	97.5	**96.6**

**Table 7:** Comparison of average computational time for testing one trial with different competing methods for subject aa.

Methods	Testing Time (ms)
FBCSP	78.8
CTFSP	143.2
DFBCSP	146.6
Fusion	23.4
STFSCSP	45.2
Class Separability	72.6
Our method (MIBIF)	156.4
Our method (PCA)	156.7

**Table 8:** The classification accuracies of the proposed CiSSA-CSP method on experimental motor imaginary EEG.

Subject	Classification Accuracy (%)				
	CSP	CiSSA + CSP	Subtime + CiSSA +CSP	Subtime + CiSSA +CSP + MIBIF	Subtime + CiSSA +CSP + PCA
S1	70.4 ± 6.1	97.5 ± 2.9	96.4 ± 4.1	93.6 ± 5.0	95.4 ± 4.5
S2	68.2 ± 10.6	87.5 ± 5.1	91.4 ± 3.0	86.1 ± 6.4	91.8 ± 3.8
S3	61.8 ± 11.9	95.4 ± 2.4	95.4 ± 4.1	95.0 ± 4.2	97.9 ± 2.5
S4	66.8 ± 9.4	85.7 ± 7.5	88.9 ± 3.9	88.9 ± 6.8	91.8 ± 5.8
S5	76.1 ± 14.8	88.6 ± 6.9	87.1 ± 10.1	87.1 ± 13.7	90.4 ± 11.3
S6	51.4 ± 10.3	80.8 ± 10.1	85.0 ± 9.0	77.1 ± 15.7	86.8 ± 10.7
S7	61.1 ± 6.2	77.1 ± 7.6	86.1 ± 8.2	78.6 ± 6.9	89.6 ± 7.8
S8	73.6 ± 6.1	90.0 ± 5.8	87.9 ± 6.1	92.5 ± 4.9	87.9 ± 7.8
S9	77.9 ± 7.1	93.2 ± 4.9	95.0 ± 4.8	91.4 ± 7.4	96.8 ± 4.3
S10	88.6 ± 9.0	92.9 ± 5.3	91.8 ± 5.8	90.7 ± 8.3	93.9 ± 5.8
S11	85.0 ± 6.0	92.1 ± 6.7	90.7 ± 5.1	91.8 ± 5.1	94.3 ± 4.5
S12	89.3 ± 7.7	93.6 ± 5.5	95.7 ± 4.4	90.7 ± 5.9	95.4 ± 4.1
S13	77.5 ± 11.2	91.1 ± 6.6	93.6 ± 5.5	90.4 ± 8.6	95.7 ± 6.0
S14	87.9 ± 4.8	90.0 ± 2.8	95.4 ± 3.4	91.8 ± 3.8	93.9 ± 5.1
S15	82.9 ± 5.8	95.7 ± 5.3	93.6 ± 5.0	90.0 ± 5.3	94.6 ± 3.0
S16	75.7 ± 9.6	92.9 ± 5.3	93.9 ± 4.1	92.5 ± 3.9	97.9 ± 3.8
S17	73.9 ± 7.0	92.1 ± 5.3	97.1 ± 2.8	92.5 ± 6.6	97.1 ± 3.8
S18	83.6 ± 5.4	85.7 ± 7.5	92.1 ± 6.3	91.1 ± 4.8	92.5 ± 4.3
S19	63.6 ± 12.5	91.1 ± 7.4	93.6 ± 4.4	88.2 ± 9.4	95.7 ± 7.1
S20	79.3 ± 4.7	95.4 ± 3.8	95.7 ± 6.0	95.0 ± 5.9	97.9 ± 3.5
Average	74.7 ± 8.3	90.4 ± 5.7	92.3 ± 5.3	89.8 ± 6.8	93.9 ± 5.5

**Table 9:** Paired *t*-test (*α* = 0.05) result for the classification accuracy on public and experimental datasets.

	CSP	CiSSA + CSP	Subtime + CiSSA +CSP	Subtime + CiSSA +CSP + MIBIF	Subtime + CiSSA +CSP + PCA
*p*-value	-	0.0000	0.0018	0.0006	0.0001

Paired *t*-test is used between two adjacent methods. For example 0.0000 is the paired *t*-test result between CSP and CiSSA + CSP methods and 0.0018 is the paired *t*-test result between Subtime + CiSSA + CSP and CiSSA + CSP methods.

**Table 10:** The highest classification accuracies and the selected feature dimension (*k*) by MIBIF and PCA for all subjects on the experimental data we recorded.

Subject	MIBIF		PCA	
	Accuracy (%)	Dimension (*k*)	Accuracy (%)	Dimension (*k*)
S1	97.5 ± 4.5	39	98.6 ± 2.5	17
S2	91.8 ± 4.5	15	93.2 ± 3.1	11
S3	97.1 ± 3.7	32	98.2 ± 2.5	8
S4	90.4 ± 6.3	17	93.9 ± 4.5	3
S5	88.2 ± 10.5	55	91.8 ± 11.6	7
S6	85.4 ± 11.1	69	90.0 ± 6.3	28
S7	87.9 ± 7.9	28	90.7 ± 5.4	14
S8	92.5 ± 4.9	9	92.9 ± 5.6	23
S9	95.4 ± 5.1	67	98.2 ± 2.5	15
S10	92.5 ± 6.2	11	95.0 ± 5.4	11
S11	93.9 ± 4.5	5	94.6 ± 4.5	14
S12	96.8 ± 3.6	47	96.4 ± 3.8	8
S13	94.3 ± 6.3	17	95.7 ± 6.0	9
S14	96.1 ± 4.9	63	95.4 ± 3.4	62
S15	95.7 ± 5.5	30	96.8 ± 3.6	14
S16	94.6 ± 4.2	45	97.9 ± 3.8	9
S17	97.5 ± 2.4	60	97.5 ± 2.9	13
S18	93.6 ± 4.1	24	93.6 ± 5.5	19
S19	95.0 ± 3.5	23	95.7 ± 7.1	9
S20	98.6 ± 3.0	36	98.6 ± 3.5	15
Average	93.7 ± 5.3		95.2 ± 4.7	

## Data Availability

The link of BCI Competition III dadasets IVa is: https://www.bbci.de/competition/iii/ (accessed on 19 September 2022).
